# Synchronous Gallstone Ileus and Bouveret’s Syndrome: A Report of Two Rare Concurrent Complications of Gallstone Disease

**DOI:** 10.7759/cureus.35672

**Published:** 2023-03-01

**Authors:** João L Pinheiro, Ana Logrado, Débora Aveiro, Maria João Ferreira, Jorge Pereira

**Affiliations:** 1 Cirurgia Geral, Centro Hospitalar Tondela-Viseu, Viseu, PRT; 2 Cirurgia Hepatobiliopancreática, Centro Hospitalar Tondela-Viseu, Viseu, PRT

**Keywords:** enterolithotomy, cholecystoduodenal fistula, bilioenteric fistula, intestinal obstruction, gastric outlet obstruction, gallstone ileus, cholelithiasis, bouveret’s syndrome

## Abstract

Cholecystoenteric fistulas occur as a result of a chronic inflammatory insult involving the gallbladder and the erosion of both its wall and a bowel segment. When the fistula develops, it creates a pathway for gallstones to migrate and cause an intestinal obstruction, known as gallstone ileus. When it obstructs the gastric outlet, a proximal variant of gallstone ileus occurs, known as Bouveret’s syndrome.

A 65-year-old man presented to the emergency department with a three-day history of epigastric and right upper quadrant pain and persistent vomiting, preceded by unintentional weight loss of 15 kg over three months. Endoscopic and complementary imaging studies identified a concurrent gastric outlet obstruction caused by a lodged gallstone in the duodenal bulb and gallstone ileus.

The patient underwent an urgent exploratory laparotomy and was submitted to an enterolithotomy and gastrolithotomy. Due to a sudden deterioration on the fourth postoperative day, he underwent an emergent re-laparotomy that found fecal peritonitis and complete dehiscence of both closures. The patient was then managed with damage control surgery. An atypical gastric resection and enterectomy of the distal ileum were performed and the patient was admitted to the intensive care unit in temporary abdominal closure (laparostomy). The patient failed to improve and died on the same day. Ultimately, the patient’s multiple comorbidities, including morbid obesity, malnutrition, and diabetes, contributed to poor tissue healing and the fatal outcome.

Gallstone ileus and Bouveret’s syndrome are two rare complications of cholecystoduodenal fistulas that have not yet been reported to occur simultaneously. Both intestinal and gastric obstruction makes the surgical approach the first-line treatment.

## Introduction

Cholelithiasis is a very common condition worldwide, more prevalent in western countries, with an incidence that can reach 15%. During their lifetimes, 20% of patients with gallstones will develop symptoms and different complications. Cholecystoenteric fistulas occur as a result of a chronic inflammatory insult involving the gallbladder and the erosion of both its wall and a bowel segment [1,2]. Due to the gallbladder’s posterior relation with the duodenum, the bilioenteric fistula develops most frequently to the duodenal bulb, creating a pathway for large gallstones, often >2.5 cm in diameter, to migrate to the intestinal lumen and result in an intestinal obstruction, known as gallstone ileus [3]. However, when the fistula develops and a gallstone obstructs the gastric outlet, a proximal variant of gallstone ileus occurs, resulting in Bouveret’s syndrome [1-3]. The presented case reports two unique complications of biliodigestive fistulas occurring at the same time in a patient with gallstone disease.

## Case presentation

A 65-year-old man presented to the emergency department with a three-day history of right upper quadrant pain, followed by persistent nausea and vomiting. The onset of symptoms was preceded by unintentional weight loss of 15 kg during the previous three months, associated with intermittent dysphagia. His past medical history included hypertension, morbid obesity (body mass index: 41 kg/m^2^), type 2 diabetes mellitus, and recently excised colonic polyps with low-grade dysplasia.

Initial blood workup showed leukocytosis of 12.3 × 10^9^/L (4.5-11.5 × 10^9^/L), lactic acid of 1.8 mmol/L (<1 mmol/L), C-reactive protein of 3.66 mg/L (<0.5 mg/dL), and hyperamylasemia of 85 IU/L (13-53 IU/L). Regarding cholestasis markers, alkaline phosphatase was 154 IU/L (25-100 IU/L) on admission, with aspartate transaminase and total Bilirubin within the normal range. Albuminemia was 2.4 g/L (3.5-5.5 g/L). The abdominal ultrasound described a partially collapsed gallbladder filled with gallstones, without wall edema or stratification, and non-dilated intrahepatic bile ducts. Additionally, multiple solid nodular hepatic lesions were found.

**Figure 1:**
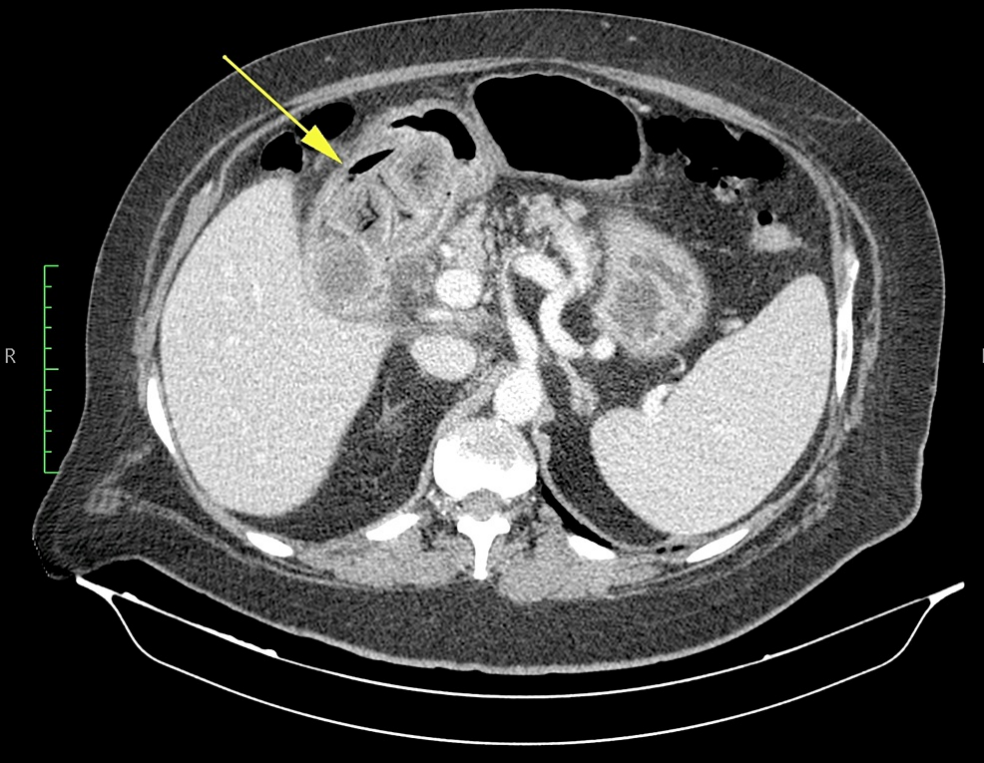
Multiple gallstones and air-fluid level inside the gallbladder (arrow).

An abdominal and pelvic computed tomography (CT) was ordered to clarify the ultrasound findings which showed a distended gastric antrum filled with heterogeneous content, in association with a thickened pyloric region and densification of the surrounding structures. The gallbladder was filled with gallstones and had an air-fluid level inside it (Figure 1). The hepatic lesions found in the ultrasound were suspicious for malignancy in the acquired CT, described as multiple solid and hypodense nodular lesions with late washout, and required further imaging studies.

A nasogastric tube was placed to empty the stomach, and the patient was admitted for further imaging workup.

An upper gastrointestinal (GI) endoscopy was performed which revealed a grade D peptic esophagitis (Los Angeles Classification) and extensive ulceration of the duodenal bulb that did not allow for further progression to the duodenum. Biopsies were taken and the patient was started on a proton pump inhibitor (PPI) infusion of esomeprazole at 8 mg/hour for 72 hours.

**Figure 2:**
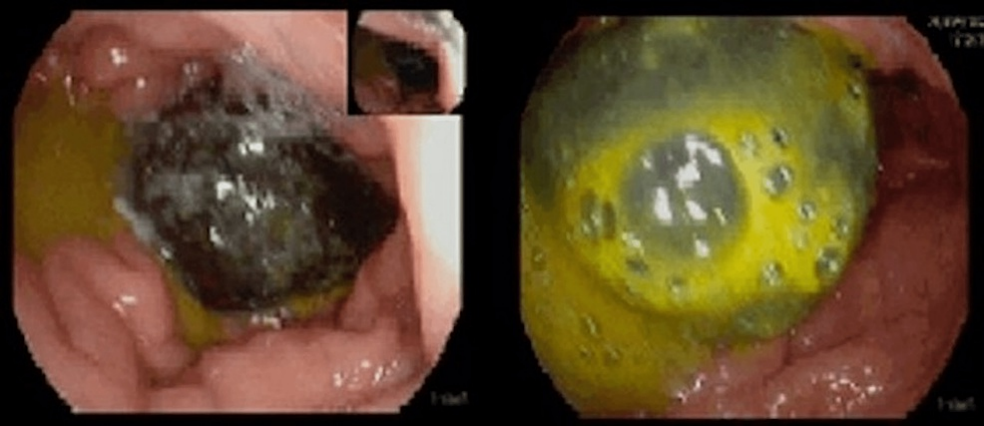
Endoscopic view of the gallstone in the duodenal bulb after PPI infusion occupying the lumen of the gastric outlet. PPI: proton pump inhibitor

After three days, the significant inflammation associated with the duodenal ulcer subsided and a second upper GI endoscopy was performed to assess the gastric outlet. A 4 cm wide gallstone was found lodged in the duodenal bulb (Figure 2). Given the stone’s large diameter, the attempts at endoscopic mechanical fragmentation failed.

**Figure 3:**
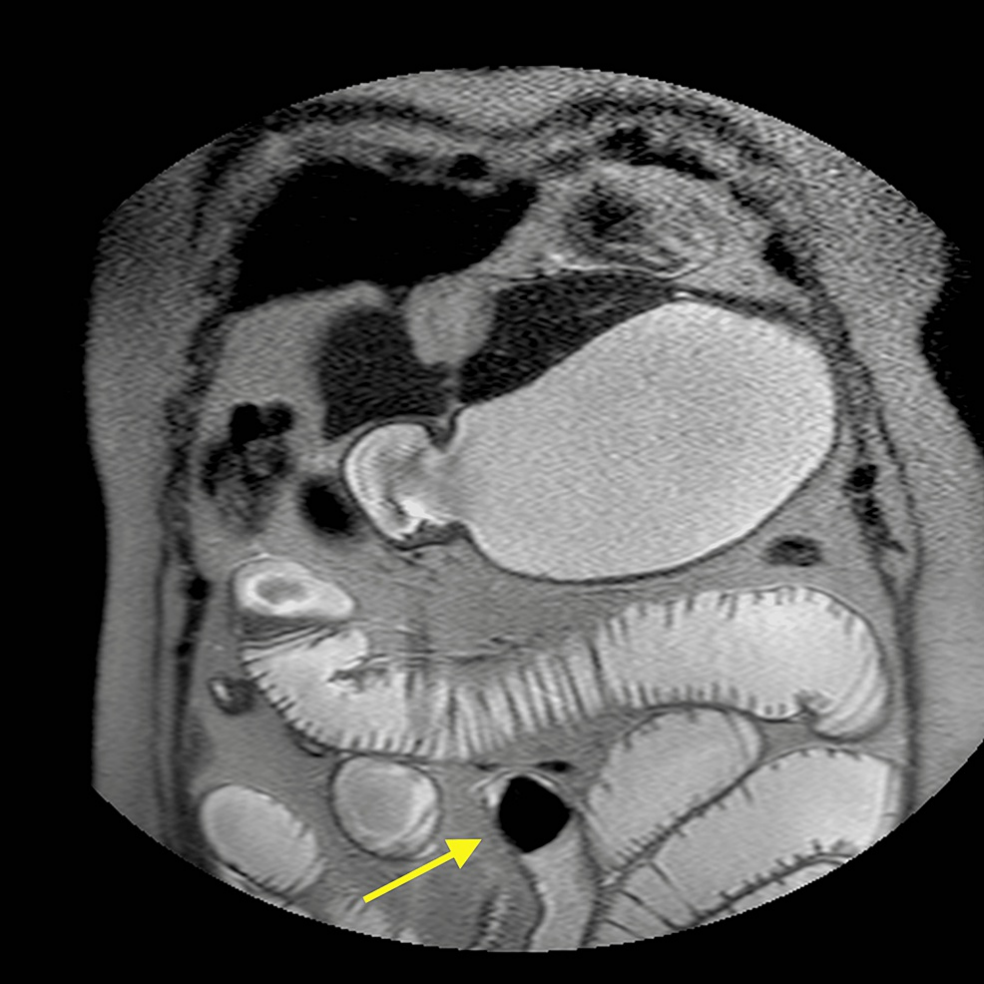
Distal migration of a gallstone (arrow) with proximal small bowel distension.

**Figure 4:**
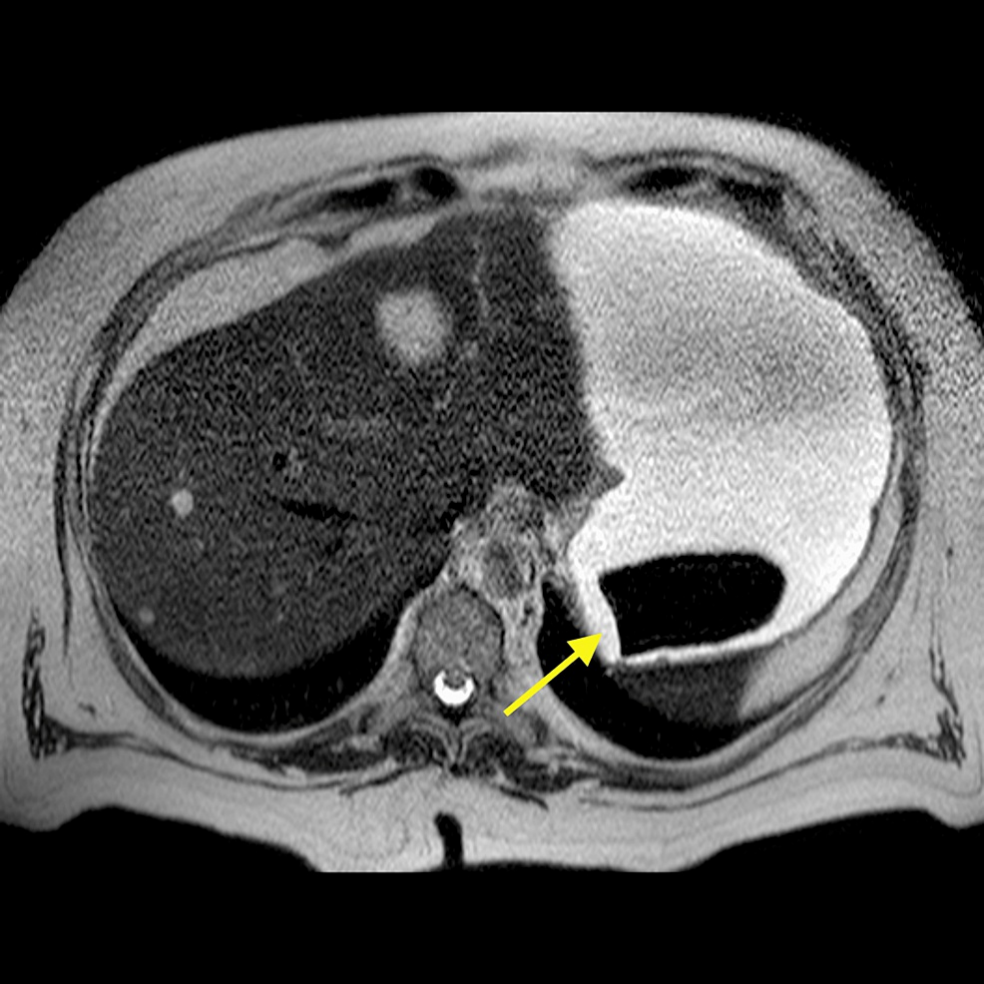
A T2-hypointense subtraction image (arrow) in the gastric lumen suggestive of proximal migration of the gallstone.

On the same day, a magnetic resonance cholangiopancreatography was done to study the hepatic nodular lesions which were confirmed to be cavernous hemangiomas. It also showed that the gallbladder was now completely empty and collapsed, suggesting a complete gallstone clearance through a bilioenteric fistula. The intestinal obstruction causing distension of the first jejunal loops along with the distal migration of a gallstone confirmed the diagnosis of a gallstone ileus (Figure 3). Moreover, in the gastric lumen, another gallstone could be identified, initially not present in the CT scan, which possibly resulted from the proximal migration of the obstructing stone (Figure 4).

**Figure 5:**
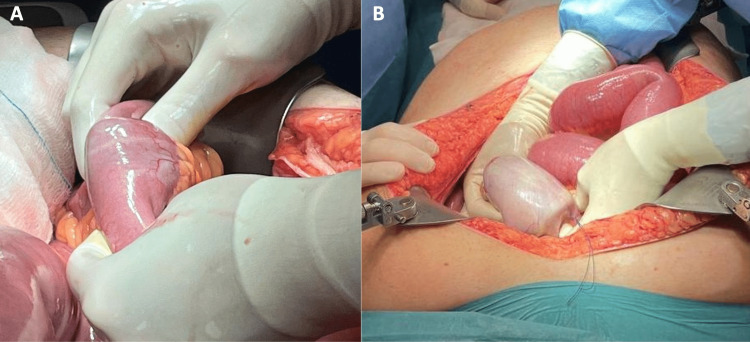
Intraoperative findings. A: A gallstone found approximately 15 cm from the ileocecal valve. B: Gastric projection of the gallstone previously causing Bouveret’s syndrome.

Because the patient had a two-site intestinal obstruction, with a diagnosis of Bouveret’s syndrome and concurrent gallstone ileus, he underwent an urgent exploratory laparotomy. A gallstone was found in the terminal ileum (Figure 5A) and a giant gallstone was visible protruding through the gastric antrum (Figure 5B).

**Figure 6:**
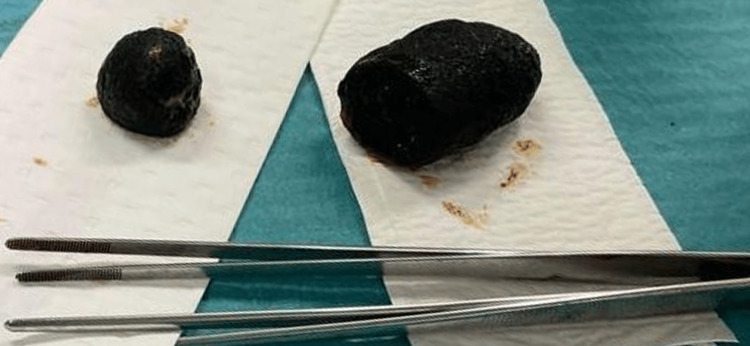
Removed gallstones: 3 cm (left) and 9.5 cm (right) of longer axis, respectively.

An enterolithotomy and a gastrolithotomy were performed to remove the dislodged gallstones (Figure 6) and were both closed with a continuous running suture. Intraoperatively, the gallbladder was collapsed and indissociable from the duodenal bulb. Both were adherent to the omentum that had migrated to the right hypochondrium.

The patient improved well during the first days after surgery, and a liquid diet was started. Despite the initial improvement, he started deteriorating on the fourth postoperative day. Broad-spectrum antibiotics were administered due to the ensuing fever and rising inflammatory markers, ultimately leading to an emergent re-laparotomy. Intraoperative findings included fecal peritonitis and complete dehiscence of both gastric and ileal closures.

Given the hemodynamic instability, the patient was managed with damage control surgery. An atypical gastric resection and a 10 cm enterectomy involving the previous ileal closure were performed and the patient was admitted to the intensive care unit in laparostomy. Despite the optimization of all resuscitation measures, the patient failed to improve and died on the same day.

## Discussion

Biliodigestive fistulas are a rare complication of cholelithiasis, occurring in up to 5% of patients with gallstones [4]. In about 60% of cases, the fistula is cholecystoduodenal and less commonly involves the stomach or colon. When patent, it enables the migration of gallstones to the gastrointestinal tract. Depending on its size and the involved digestive portion, it can cause an intestinal obstruction [3,4].

The terminal ileum is the most common place of gallstone impaction which defines the classic gallstone ileus. Usually, it involves stones larger than 2.5 cm in diameter [5].

Bouveret’s syndrome is considered an uncommon variant of gallstone ileus. It accounts for fewer than 3% of all cases and consists of a gastric outlet obstruction due to an impacted gallstone [3]. This rare complication is more frequent in frail older women, usually with multiple comorbidities [2,5,6]. The presented case refers to a 65-year-old male patient who, although not elderly, was an older adult with multiple cardiovascular risk factors, which contributed to his overall poor clinical status.

The clinical presentation of biliodigestive fistulas is usually non-specific [7]. It can range from nausea, weight loss, or occasional low-intensity biliary colic to an acute-onset gastric outlet obstruction with persistent vomiting [2]. In the presented case, the intermittent gastric and ileal obstruction due to the occasional gallstone dislodgement made the diagnosis challenging and the clinical presentation very insidious. The blood workup can show elevated cholestasis markers and elevated serum amylase [5], which was also consistent with our patient’s biochemical panel.

Ultimately, when Bouveret’s syndrome develops, upper endoscopy remains an examination of significant diagnostic and therapeutic value with various techniques available such as laser, shockwave, or electrohydraulic lithotripsy to fragment larger gallstones [8]. Further imaging studies, such as abdominal CT, are also crucial to identify the level and cause of intestinal obstruction. Plain abdominal radiography has a sensitivity for the diagnosis of gallstone ileus of up to 70%. However, the pathognomonic Rigler triad, defined as radiologic findings of pneumobilia, small bowel obstruction, and ectopic calcified gallstones, only meets its full criteria in about half of the cases [7].

Concerning treatment options, endoscopic extraction or lithotripsy should be considered the first-line approach, given the poor performance status of most patients with this condition. However, it is only so when there are no other documented sites of obstruction and the size of the gallstone enables its manipulation. The endoscopic retrieval of gallstones or their fragmentation is generally successful when the diameter is less than 3 cm, limiting its utility in the presented case [9]. Further, one drawback of the endoscopic treatment of large proximal impacted gallstones is the possibility of distal migration of its fragments resulting in secondary gallstone ileus [2,4]. Other complications associated with endoscopic treatment include bowel injury and the risk of perforation [10]. Our patient was neither a candidate for endoscopic treatment nor extracorporeal shock-wave lithotripsy, given his unique clinical presentation with a concurrent proximal and distal obstruction along with morbid obesity.

Regarding surgical treatment, the most agreed-upon procedure is an enterolithotomy or, if feasible, a gastrolithotomy after mobilization of the gallstone to the gastric lumen. Performing a cholecystectomy with fistula breakdown in the acute onset is a procedure of high complexity due to the local inflammatory status and is still a matter of debate whether it is beneficial for already debilitated patients [5]. In the case reported, there was the need for two lithotomies because the duodenal ulceration would not allow for the ileal gallstone to pass to the stomach and be removed through a single incision.

Ultimately, the patient’s multiple comorbidities, including morbid obesity, malnutrition, and diabetes, contributed to poor tissue healing and the fatal outcome.

## Conclusions

To our knowledge, gallstone ileus and Bouveret’s syndrome are two rare complications of cholecystoduodenal fistulas that have not yet been reported to occur concurrently. Both intestinal and gastric obstruction makes the surgical approach the first-line treatment. When possible, the gallstones should be mobilized to the stomach and removed through a single incision. However, the duodenal inflammatory status might preclude this option.
